# Supplementary material for: Gender effect in survival after out-of-hospital cardiac arrest: A nationwide, population-based, case-control propensity score matched study based Korean national cardiac arrest registry
Source: PLoS One. 2022 May 11;17(5):e0258673. doi: 10.1371/journal.pone.0258673 (PMC9094503; doi:10.1371/journal.pone.0258673)
Supplement: Attachment — Submitted filename: Response to Reveiwer2.docx [file pone.0258673.s001.docx]

**Reviewer #2: Thank you for the opportunity to review your manuscript entitled "Gender effect in survival after out-of-hospital cardiac arrest: a nationwide, population-based, case-control propensity score matched study based Korean National Cardiac Arrest registry".**

Dear reviewer.

Thank you for giving us the opportunity to submit a revised draft of the manuscript. We appreciate the effort you and you have devoted to providing your valuable feedback on the manuscript. We are thankful to you for your insightful comments on the paper. We have been able to incorporate changes to reflect the suggestions provided by you.

Here is a point-by-point response to your comments and concerns.

**First of all, I would like to suggest you specify the total word count at the beginning of the manuscript and insert line numbers to make it easier to review and to correct any mistake.**

Thank you

**Specific comments:
Abstract
-Please remove the comma between "OR" and the value. Moreover, you should write "95% CI" before the interval and at the end the p-value inside the brackets when you present your results.

Materials and Methods
-Interesting description of your EMS setting, thank you.**

Thank you for your kind compliment.

**-Has this study an approval from the ethics committee? If yes, you should specify.**

Thank you for your advice. This study was approved by the institutional review boards of the participating institutions (IRB number: 2021-05-005), and the need for informed consent was waved. We additionally mention this in front of method section as bellow

*This study was approved by the institutional review boards of the participating institutions (IRB number:* 2021-05-005*), and the need for informed consent was waved.*

**Results
-Consider rephrasing: "From January 1, 2009, to December 31, 2016 a total of 214,954 patients with OHCA were identified
from the OHCA database".**

Thank you for your advice, we had changed sentence as below

***“Total 214,954 patients of OHCA were identified in this study from January 1, 2009, to December 31, 2016.”***

**-The description of inclusion and exclusion flow chart is not very clear. Considering the numbers, it seems that no patient excluded from the study has more than one excluding criteria (i.e.,** **under 18 years old with arrest due to trauma). Moreover, you should specify what you mean with "arrest of non-cardiac etiology", because also an arrest due to trauma has not cardiac etiology.**

Thank you for your advice. We had excluded patients step by step. Therefore, there might be some patients who had more than one exclusion criteria. However, these patients had been banned in the previous step already. For example, a patient under 18 years old with arrest due to trauma had excluded in exclusion of patient in arrest due to trauma. We had added these contents in figure 1 legend as below.

*“**We had excluded patient step by step from top to above. “*

**-Consider changing inclusion and exclusion flow chart (Fig.1): you should separate the chart about 18-44 years and >=55 years in the main flow chart you should only specify the number of female and male of the 94,160 patients included.**

I totally agree with your point. We had changed figure 1 as following and clarify the separation of patients.

**Discussion
-Consider changing every semicolon to punctuation in the paragraph "A previous study suggested that OHCA was more likely to occur at home in female patients (...) the presence of ventricular fibrillation is known to show a better prognosis than that of asystole or pulseless electrical activity, according to the latest studies".**

I agree with your point. We had changed above paragraph as below

*A previous study suggested that OHCA was more likely to occur at home in female patients, who were admitted with a negative prognosis [27]. Female patients tended to have a low survival rate due to less prehospital resuscitation efforts and the social norms of a community in attempting chest compression or defibrillation in women [3]. Female patients tended to have a lower initial shockable rhythm (ventricular fibrillation or pulseless ventricular tachycardia) than male patients. Moreover, the presence of ventricular fibrillation is known to show a better prognosis than that of asystole or pulseless electrical activity, according to the latest studies [27].* **–**

**Add a punctuation at the end of the sentence "In menopausal women (over 55 years old), the results were similar to the all age group analyses".**

I agree with your point. To clarify the sentence, we had changed sentence as below.

*In menopausal women (over 55 years old), the results were similar to analysis result in all age group.*

Thank you for your kind comments and advice. Our manuscript have improved due to your thoughtful comments.

We look forward to hearing from you in due time regarding our submission.
